# Prevention of AMI Induced Ventricular Remodeling: Inhibitory Effects of Heart-Protecting Musk Pill on IL-6 and TNF-Alpha

**DOI:** 10.1155/2017/3217395

**Published:** 2017-03-06

**Authors:** Wei Cen, Zhiliang Chen, Ning Gu, Ralph Hoppe

**Affiliations:** ^1^Nanjing University of Chinese Medicine, Nanjing 210023, China; ^2^The Third Affiliated Hospital of Nanjing University of Chinese Medicine, Nanjing 210001, China; ^3^Ganzheitliches Gesundheits Zentrum, 34305 Hessen, Germany

## Abstract

Heart-Protecting Musk Pill (HMP) is a Traditional Chinese Medicine (TCM) that has been used for the prevention and treatment of coronary heart disease in clinic. The current study investigated the effect of HMP on the concentrations of interleukin-6 (IL-6) and tumor necrosis factor-alpha (TNF-*α*) and observed the relationship between level changes of inflammatory cytokines and ventricular remodeling in rats with acute myocardial infarction (AMI). Animal models of AMI were made by coronary artery ligation in Sprague-Dawley (SD) rats. AMI rats showed increased levels of IL-6 and TNF-*α*. Treatment with HMP decreases IL-6 and TNF-*α* concentrations in rats with AMI. Histopathological and transmission electron microscopic findings were also essentially in agreement with biochemical findings. The results of our study revealed that inflammatory cytokines IL-6 and TNF-*α* induce cardiac remodeling in rats after AMI; HMP improves cardiac function and ameliorates ventricular remodeling by downregulating the expression of IL-6 and TNF-*α* and further suppressing the ultrastructural changes of myocardial cells.

## 1. Introduction

Heart-Protecting Musk Pill (HMP), a multiherbal medicine developed according to Traditional Chinese Medicine (TCM) theory, is widely adopted in clinical practice to treat coronary heart disease. In HMP,* Moschus* is the monarch drug with effect of resuscitation, analgesia, and removing obstruction in collaterals. Panax Ginseng supplements and strengthens vital Qi [1] which is one of the fundamental substances maintaining life activities; the pungent and warm natured Styrax Liquidus helps the monarch drug to enhance resuscitation; both of them are minister drugs in this pill. Secretio Bufonis, Bezoar, and Cinnamon serve as assistant drugs in HMP. Secretio Bufonis induces resuscitation and relieves pain, while Bezoar refreshes heart and Cinnamon invigorates warmly heart Yang [2–5]. Borneol as the guide drug helps the monarch drug in resuscitation and relieving pain. The synergistic actions of all these drugs have the effect of warming up the heart Yang, activating blood circulation, eliminating phlegm, relieving pain, benefiting cardiac Qi, and protecting heart. By numerous animal experiments and clinical studies, it is proved that HMP is capable of dilating coronary artery, reducing the myocardial infarcted size, preventing ventricular reconstitution, inhibiting the inflammation of blood-vessel wall, and stabilizing atherosclerosis plaques [6, 7].

Acute myocardial infarction (AMI) is regarded as “the first killer” to human health. Pathologic ventricular remodeling following AMI mainly refers to the process of changes in size, shape, structure, and function of heart, including ventricular dilatation in infarcted zones and deterioration in cardiac function resulting from myocardial necrosis, myocardial fibrosis, and expansion of ventricular wall. Although some studies and clinical applications of HMP already got involved in the intervention of ventricular remodeling, to the best of our knowledge, so far, no research has ever reported that the interventional approach is mainly through inhibiting the inflammatory response. The present study was therefore aimed at exploring the possible mechanism of HMP on prevention of AMI induced ventricular remodeling by observing the correlation of cardiac remodeling with IL-6 and TNF-*α* expression as well as the intervention effect of HMP on IL-6 and TNF-*α*.

## 2. Materials and Methods

### 2.1. Drugs and Reagents

Heart-Protecting Musk Pill was the product of Shanghai Hutchison Pharmaceuticals Co., Ltd (batch number 09050201). Simvastatin tablets were the product of Hangzhou Merck pharmaceutical Co., Ltd (batch number J20040032). ELASA test kit was purchased from Shanghai Bluegene Biotech CO., Ltd (batch number 20100315).

### 2.2. Experimental Animals

A total of 70 healthy, male SD rats, weighing 319 ± 21 g, were supplied by the Experimental Animal Center of Nanjing University of Chinese Medicine with the approval number of SYXK (SU) 2012-0042. They were provided with unlimited access to food and tap water.

### 2.3. Instruments

ALC-V8 Animal artificial respiration machine was purchased from Shanghai Alcott Biotech Co., Ltd. Animal electrocardiograph was purchased from Shanghai Kohden Medical Electronic Instrument Corporation. Electronic Precision Balance was purchased from Shanghai Precision & Scientific Instrument Co., Ltd. Biological signal acquisition and processing system was purchased from Chengdu instrument factory. JEM-1200EX Transmission electron microscope was purchased from Japanese Electronics Co., Ltd.

### 2.4. Experimental Design

Experimental rats were randomized into 6 groups. In addition to the normal control group (group A) including 8 healthy SD rats without any operation, the other 5 groups were established as AMI models: SD rats were anaesthetized with ketamine. After linking with respirator, the left anterior descending coronary arteries of rats were ligated. The standard of a successful model preparation was as follows: part of anterior wall myocardium in left ventricular turned into pale; ventricular wall motion decreased; meanwhile electrocardiogram (ECG) displayed ST segment elevation. Eight rats in the sham-operated group (group B) underwent similar surgical process but their coronary arteries were not ligated. After operation, rats were given daily intramuscular injection of 40000-unit penicillin for 5 days to prevent infection. The successfully created rats model was randomly assigned to AMI group (group C) including 9 rats, HMP equivalent dosage group (group D) including 9 rats, HMP high dosage group (group E) including 9 rats, and Simvastatin group (group F) including 9 rats. From the second day after operation, rats in groups A, B, and C were fed with normal saline of 10 mL·kg^−1^·d^−1^, group D with HMP of 14 mg·kg^−1^·d^−1^, group E with HMP of 28 mg·kg^−1^·d^−1^, and group F with Simvastatin of 2 mg·kg^−1^·d^−1^. The intragastric administration was done in this process for 2 weeks (note: experimental equivalent dosage for rats is calculated by the formula that converts daily dosage of adult (g/kg) into that of rat by multiplying a conversion coefficient 6.25 [8] and twice the equivalent dosage was regarded as high dosage).

### 2.5. Hemodynamic Monitoring

Rats were anesthetized after 2 weeks of treatment. Following common carotid artery intubation, a catheter with heparin-saline was introduced into the left ventricular to monitor LVESP and LVEDP through a pressure sensor and a data acquisition system. LV + *dp*/*dt*_max_ and LV − *dp*/*dt*_max_ were then calculated by differentiator. All these signals were recorded in the multilead physiological recorder when they were stable.

### 2.6. Determination of TNF-*α* and IL-6 Values in Plasma

3–5 mL blood from right common carotid artery was collected with anticoagulants added and 2 hours on standing. After being centrifuged at 3000 r/min for 15 min, ELISA was applied to determine the plasma TNF-*α* and IL-6 levels.

### 2.7. Determination of TNF-*α* and IL-6 Values in Myocardial Tissue

Part of left ischemia myocardium tissue was weighted and mixed with normal saline at a weight/volume ratio of 1 : 10 (g/mL) into 10% tissue homogenate. After centrifugation at 3000 r/min for 20 min, the contents of TNF-*α* and IL-6 in the filtrating suspensions were detected by ELISA to assess the effect of HMP on cytokine production.

### 2.8. Histopathology of Myocardium

Cardiac specimens from all the groups were washed immediately with saline and then fixed in 10% buffered neutral formalin solution. After that, the tissues were processed by embedding in paraffin. Then, the tissues were cut in sequential sections of 5 *μ*m and stained with hematoxylin-eosin (H&E) and Masson trichrome for study.

### 2.9. Ultrastructural Observation on Ischemia Myocardium Areas

Myocardial tissues of about 2 mm in length were cut from both infarcted and noninfarcted regions of rats and immediately fixed with 4% glutaraldehyde and kept at 4°C for 12 h. After fixation, wash, electron staining, dehydration embedding, and specimens preparation, ultramicrostructures of cells were observed via JEM-1200EX transmission electron microscopy.

### 2.10. Statistical Analysis

The experimental data were analyzed for statistical significance by one-way Analysis of Variance (ANOVA) using the software SPSS16.0. Results were expressed as mean ± standard deviation. Differences were considered statistically significant at a *P* value of <0.05.

## 3. Results

### 3.1. Basic Information of Rats

Two rats in group C and 1 rat in group D died of AMI, and 1 rat in group F died of wound infection.

### 3.2. Effect of HMP on Hemodynamics

Complete experimental variables were obtained in rats which were from group A, group B, group C, group D, group E, and group F. Compared to groups A and B, LVESP and LV ± *dp*/*dt*_max_ markedly decreased; LVEDP markedly increased (*P* < 0.05 or *P* < 0.01) in groups C, D, E, and F. As expected, all these data improved greatly in 3 treatment groups D, E, and F (*P* < 0.05 or *P* < 0.01). No obvious difference between group D and group F on indexes of LVESP and LV + *dp*/*dt*_max_ (*P* > 0.05) was observed. However, the differences had statistical significance when the two groups were compared with group E (*P* < 0.05). LVEDP and LV − *dp*/*dt*_max_ were insignificantly different among the 3 experimental drug-treated groups. Hemodynamic indices are summarized in Table 1. HMP treated rats showed significant effects compared to model rats with AMI.

### 3.3. Effect of HMP on IL-6 and TNF-*α* Levels

IL-6 and TNF-*α* concentrations in myocardial tissue and plasma are shown in Table 2. In comparison to groups A and B, IL-6 and TNF-*α* values markedly increased (*P* < 0.05 or *P* < 0.01) in groups C, D, E, and F. Concentrations of IL-6 and TNF-*α* were significantly lower in groups D, E, and F compared with group C (*P* < 0.05 or *P* < 0.01). There was no remarkable differences observed in IL-6 and TNF-*α* expression between group E and group F (*P* > 0.05), whereas the differences were significant between the two groups versus group D (*P* < 0.05).

The results showed that HMP decreases considerably IL-6 and TNF-*α* in AMI model rats. HMP consists of seven herbs including Moschus, Panax Ginseng, Venenum Bufonis, Cortex cinnamomi, Styrax, Calculus Bovis, and Borneol. Modern pharmacology studies found that the component drugs of HMP were effective in antiplatelet aggregation, strengthening heart, vasodilatation, reducing inflammation, and analgesia [9–12]. Furthermore, some studies found that HMP can diminish the myocardial infarction areas in AMI rats model, promote the angiogenesis, and improve cardiac function of AMI patients [7, 13]. As a highly valued traditional medicine, Moschus is used extensively for the treatment of cardiocerebrovascular diseases. The research by Wang et al. suggests that treatment with muscone which is the main ingredient of Moschus suppresses the expression levels of TNF-*α*, IL-1*β*, TGF-*β*1, and NF-*κ*B [14]. Enomoto et al. reported that the compounds isolated from Venenum Bufonis have inhibitory effects on IL-6 [15]. Lee and colleagues demonstrated various therapeutic effects of Ginseng and Ginseng extract [16–18]. Ginsenoside is extract of Panax Ginseng. Zhu et al. observed downregulation by Ginsenoside Rb1 (GRb1) of both IL-6 and TNF-*α* after GRb1 administration [19]. The study by Kim et al. showed that Transgenic Panax Ginseng inhibits the production of TNF-*α*, IL-6, and IL-8 as well as COX-2 expression [20]. These might partially explain the mechanism of why HMP decreases IL-6 and TNF-*α* in rats with acute myocardial infarction.

### 3.4. Effect of HMP on Heart Histopathology

**Figure 1:**
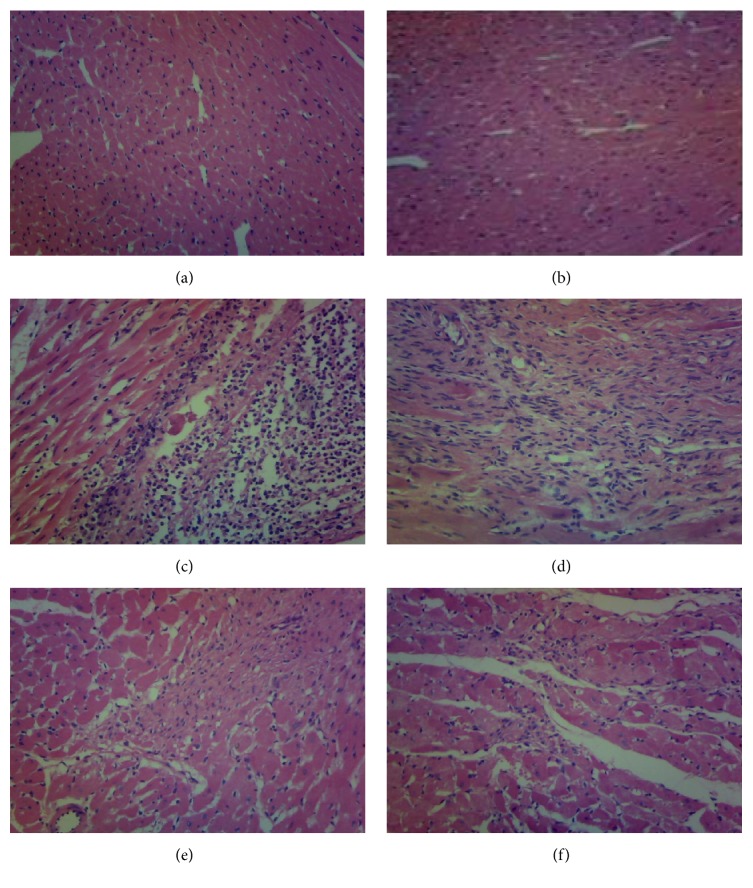
(a–f) Histopathological observations of the heart. (a) Normal control rat. (b) Sham-operated rat. (c) AMI rat. (d) HMP equivalent dosage treated rat. (e) HMP high dosage treated rat. (f) Simvastatin treated rat.

Hearts from different group showed different characteristic patterns in H&E stain. The control group and sham-operated group showed normal architecture of myocardium (Figures 1(a) and 1(b)). The rats treated with HMP high dosage also revealed normal architecture of heart without obvious damage (Figure 1(e)). But the model group of AMI rats showed separation of cardiac muscle fibers and necrosis with inflammatory cells in the H&E stained sections (Figure 1(c)). Treatment with HMP equivalent dosage revealed mild separation of cardiac muscle fibers with little inflammatory cells infiltration (Figure 1(d)).

### 3.5. Effect of HMP on Myocardial Fibrosis

**Figure 2:**
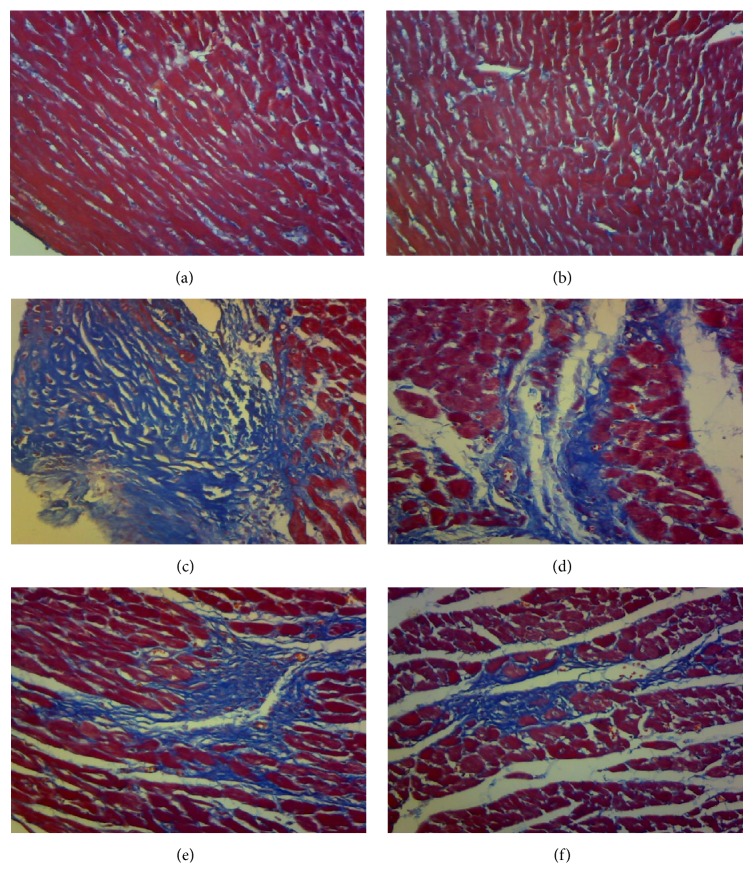
(a–f) Masson's staining for myocardial fibrosis detection. (a) Normal control rat. (b) Sham-operated rat. (c) AMI rat. (d) HMP equivalent dosage treated rat. (e) HMP high dosage treated rat. (f) Simvastatin treated rat.

Figures 2(a)–2(f) show the effect of HMP on myocardial fibrosis in normal and AMI rats. Overall, images of normal control rats and sham-operated rats revealed no obvious collagen hyperplasia (Figures 2(a) and 2(b)); the fibrotic area was significantly increased in the rats with AMI (Figure 2(c)); treatment with HMP and Simvastatin attenuated myocardial fibrosis and collagen deposition considerably (Figures 2(d)–2(f)). The collagenous fibrous tissue was stained blue with the Masson trichrome stain. The results revealed that HMP prevents myocardial fibrosis in rats with AIM.

### 3.6. Effect of HMP on Myocardial Ultrastructure

**Figure 3:**
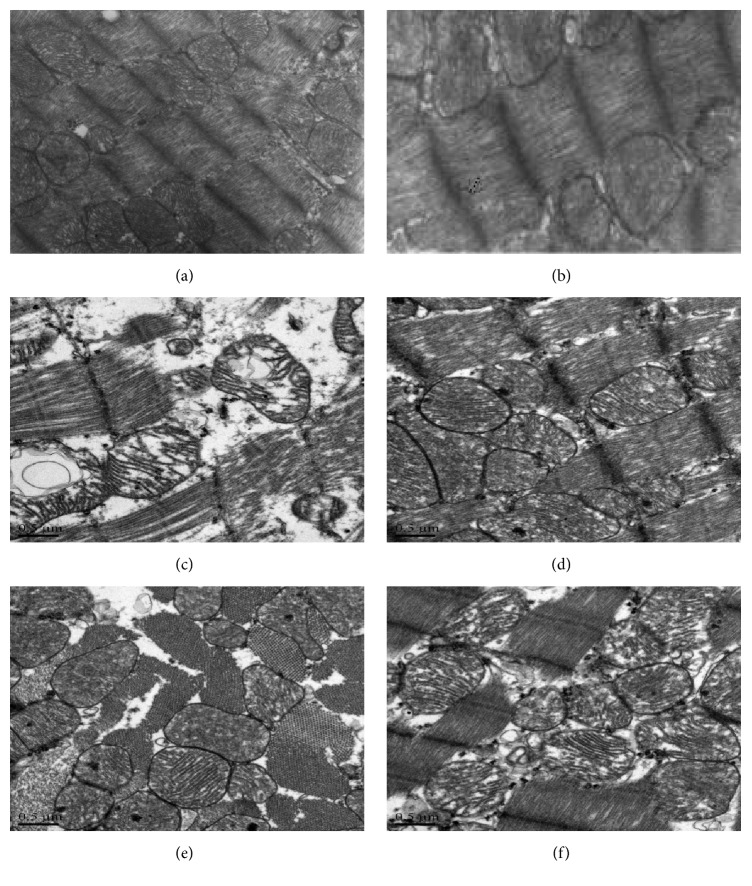
(a–f) TEM observations of the heart. (a) Normal control rat. (b) Sham-operated rat. (c) AMI rat. (d) HMP equivalent dosage treated rat. (e) HMP high dosage treated rat. (f) Simvastatin treated rat.

From TEM observation of normal control group and sham-operated group, we can see that the cardiac myocytes were arranged tightly, the sarcolemmas were integrated and with clear bands, bright and dark bands of paralleled myofilaments looked apparent, and the nuclei were oval and located at the cell center. Additionally, abundant mitochondria arranged along myofibril, double membrane structure cristae were visible and in orderly arrangement, chromatin distributed evenly. There was no blood-vessel expansion, no infiltration of inflammatory cells, no edema, and no hyperemia in intercellular substance (Figures 3(a) and 3(b)).

In model group of AMI rats, part of myocardial myofilament ruptured and exposed. Mitochondria swelled as balloon shape with confused arrangement; cristae were disrupted. Capillaries in intercellular substance dilated seriously with destroyed endothelium and red blood cells leakage. Meanwhile, myocardium necrosis, inflammatory cells infiltration, and local dissolution of myofilaments existed, and myofibers contents increased with chaotic and loose arrangement (Figure 3(c)).

Myocardial myofibril of three treatment groups was in good arrangement, intercalated discs arranged in perfect order, parts of mitochondria cristae were lightly disrupted, and sarcoplasmic reticulum expanded in mild degree. Myofilaments were well arranged, but some of them were broken. There were a little collagen hyperplasia and a little inflammatory cells infiltration (Figures 3(d)–3(f)).

## 4. Discussion

The key findings of our study were as follows: (1) HMP improved hemodynamic parameters; (2) HMP reduced myocardial fibrosis; (3) HMP exerted anti-inflammation effects by decreasing IL-6 and TNF-*α* expression; (4) HMP protected myocardial ultrastructure. To the best of our knowledge, this is the first study carried out on the preventive effects of HMP on pathologic ventricular remodeling through observing the relationship between inflammatory cytokines levels and cardiac remodeling.

Our experimental research showed that, compared with control and sham operation group, LVESP and LV ± *dp*/*dt*_max_ decreased significantly, and LVEDP, left ventricular weigh, and left ventricular weight index increased significantly 2 weeks after AMI in AMI group and the three drug-treated groups. The results demonstrated that infarction area hypertrophy obviously increased ventricular cavity and decreased cardiac function that occurred after AMI. Electron microscope further showed obviously myocardial ultrastructural changes and ventricular reconstitution after AMI. At the same time, the contents of IL-6 and TNF-*α* in plasma and myocardial tissue of AMI group and three treatment groups are elevated. These data indicated that IL-6 and TNF-*α* play an important role of inducing ventricular remodeling after AMI, as was reported in literatures [21–23]. In our experiment, after two weeks of HMP treatment, the IL-6 and TNF-*α* contents in HMP groups compared to AMI group declined obviously; meanwhile LVESP and LV ± *dp*/*dt*_max_ significantly increased and LVEDP decreased obviously. Under TEM, clearer myocardial ultrastructure, less myocardial fibrosis, and inflammatory reaction can be observed. These results indicated that a significant improvement of heart function and the inhibition of the cardiac reconstitution of rats with AMI occurred after HMP treatment, and this process might be realized partially by IL-6 and TNF-*α* reduction. HMP had similar or even better therapeutic efficacy than its positive control medicine Simvastatin, which might be related to the unique effect of Qi benefiting heart protection of HMP.

In summary, inflammatory cytokines IL-6 and TNF-*α* could induce cardiac remodeling after AMI, and HMP could reduce the levels of IL-6 and TNF-a, further decrease collagen positive area, and improve left ventricular function. This suggests that suppressing the inflammatory response might therefore be the mechanism of HMP for preventing the ventricular reconstruction of postmyocardial infarction. And, in the medication dose range, the experimental results of high dosage of HMP were better than those of equivalent dosage. Our study provides evidence for the cardioprotective effect of HMP in AMI rats.

## Figures and Tables

**Table 1:** Hemodynamic indexes of rats $\left(\overset{-}{X}\pm S\right)$.

Groups	*n*	LVESP (mmHg)	LVEDP (mmHg)	LV + *dp*/*dt*_max_ (mmHg/s)	LV − *dp*/*dt*_max_ (mmHg/s)
A	8	132.59 ± 12.19	5.37 ± 0.97	5607.5 ± 804.64	4745.0 ± 685.64
B	8	133.19 ± 9.45	5.25 ± 0.89	5388.4 ± 377.47	4766.0 ± 570.98
C	7	89.65 ± 6.06^##^	7.57 ± 4.87^##^	3050.9 ± 507.65^##^	2843.9 ± 631.72^##^
D	8	100.80 ± 5.52^##▲^	6.95 ± 0.19^#▲^	4257.7 ± 413.87^##▲▲^	3758.2 ± 347.50^##▲▲^
E	9	109.71 ± 5.28^##▲▲★^	6.81 ± 0.32^#▲^	4799.3 ± 450.70^##▲▲★^	4173.9 ± 371.45^#▲▲^
F	8	99.77 ± 4.39^##▲^	6.88 ± 0.22^#▲^	3960.1 ± 351.86^##▲▲^	3732.2 ± 393.50^##▲▲^

^#^ *P* < 0.05, ^##^*P* < 0.01  versus group A; ^▲^*P* < 0.05, ^▲▲^*P* < 0.01  versus group C; ^★^*P* < 0.05 versus group F.

**Table 2:** IL-6 and TNF-*α* data of rats ($\overset{-}{X}\pm S$).

Groups	*n*	IL-6 in tissue ng/L	TNF-*α* in tissue ng/mL	IL-6 in plasma ng/L	TNF-*α* in plasma ng/mL
A	8	5.43 ± 0.54	5.60 ± 3.09	40.91 ± 8.21	0.71 ± 0.25
B	8	5.48 ± 0.72	6.15 ± 4.32	42.35 ± 9.80	0.72 ± 0.19
C	7	8.39 ± 0.56^##^	28.43 ± 7.10^##^	122.57 ± 15.90^##^	1.61 ± 0.23^##^
D	8	7.61 ± 0.83^##▲^	20.33 ± 7.80^##▲^	91.31 ± 18.23^##▲▲★★^	1.31 ± 0.21^##▲▲★★^
E	9	6.92 ± 0.70^##▲▲★★^	13.88 ± 7.80^##▲▲★★^	71.85 ± 8.36^##▲▲^	1.07 ± 0.14^##▲▲^
F	8	6.69 ± 0.52^##▲▲★^	12.85 ± 4.23^#▲▲★^	60.57 ± 13.45^##▲▲^	0.96 ± 0.15^##▲▲^

^#^ *P* < 0.05, ^##^*P* < 0.01  versus group A; ^▲^*P* < 0.05, ^▲▲^*P* < 0.01  versus group C; ^★^*P* < 0.05, ^★★^*P* < 0.01  versus group F.
